# Sobel neural network for EEG-based major depressive disorder screening

**DOI:** 10.3389/fpsyt.2025.1667107

**Published:** 2025-11-07

**Authors:** Hui Yang, Yu Ye

**Affiliations:** 1 Computer School (Huangshi Key Laboratory of Computational Neuroscience and Brain-Inspired Intelligence), Hubei Polytechnic University, Huangshi, China; 2 Department of Radiology, The Central Hospital of Huangshi City, Huangshi, China

**Keywords:** classification, depression, EEG, Sobel, neural network

## Abstract

Early and objective screening for major depressive disorder (MDD) is crucial, with electroencephalography (EEG) offering significant potential. However, developing accurate automated tools requires architectures adept at capturing subtle, discriminative spatiotemporal features in EEG signals. This paper introduces the Sobel Network, a novel neural architecture designed specifically for EEG-based MDD screening, namely, identifying MDD patients from healthy controls (HC). Unlike approaches using Sobel operators solely for preprocessing, the Sobel Network integrates Sobel-inspired operations intrinsically within its convolutional layers, enabling end-to-end learning of features emphasizing gradient patterns and edge-like information highly relevant to depression biomarkers in EEG. We evaluate the Sobel Network on a publicly available EEG dataset from the Hospital of Universiti Sains Malaysia (HUSM). This dataset comprises 34 patients diagnosed with MDD (17 men; mean age, 40.3 ± 12.9 years) and 30 healthy controls (HC; 21 men; mean age, 38.2 ± 15.6 years). The results demonstrate that the proposed architecture significantly outperforms other deep learning models in key metrics including accuracy (achieving 98.67%), sensitivity (99.18%), and specificity (98.10%). The Sobel Network presents a promising avenue to improve the accuracy and robustness of automated EEG-based depression screening tools, offering practical impact for clinical decision support.

## 1 Introduction

Major depressive disorder (MDD), a pervasive and debilitating mental health disorder, affects over 300 million individuals worldwide, imposing substantial burdens on personal well-being, healthcare systems, and societal productivity (1). Its insidious onset and heterogeneous clinical presentation often leads to underdiagnosis or delayed intervention, exacerbating long-term outcomes. Early and accurate screening is therefore critical to enabling timely therapeutic interventions, yet current clinical practices remain heavily reliant on subjective self-report scales and qualitative clinical interviews (2). These methods are prone to bias, recall errors, and variability across raters, highlighting an urgent need for objective, biologically grounded screening tools specifically for MDD. It is important to note that depression encompasses multiple subtypes (e.g., persistent depressive disorder, seasonal affective disorder), but this work focuses exclusively on MDD screening.

Electroencephalography (EEG) has emerged as a promising tool for objective depression assessment due to its non-invasiveness, high temporal resolution, and sensitivity to neural dynamics. Depression-related neurophysiological abnormalities—such as altered frontal alpha asymmetry, disrupted connectivity, and aberrant synchronization—often manifest as subtle spatial gradients and edge-like transitions in multi-channel EEG topography. Recent EEG analytics highlight the value of network and complexity approaches, with studies showing that domain-specific connectivity metrics capture brain network alterations in ADHD (3), spectral coherence identifies emotion regulation strategies (4), and frequency-specific complexity discriminates maladaptive rumination (5). Most pertinent to this work, EEG-derived network analysis predicts rTMS outcomes in MDD (6), and functional connectivity patterns link to specific depressive symptoms as treatment biomarkers (7). These features reflect localized disruptions in neural circuitry and inter-regional communication, hallmarks of MDD pathology. The sensitivity of Sobel operators to such spatial gradients makes them particularly suitable for capturing these depression-specific biomarkers.

However, translating raw EEG data into reliable diagnostic markers remains challenging. EEG signals are inherently noisy, non-stationary, and high-dimensional, with subtle discriminative patterns often obscured by artifacts (e.g., muscle movements, eye blinks) and inter-individual variability. Traditional analytical approaches, which rely on handcrafted features such as spectral power and coherence, struggle to capture the complex spatiotemporal dynamics underlying depression-related neural activity, thereby limiting their sensitivity and specificity.

In recent years, deep learning has revolutionized EEG analysis by enabling end-to-end learning of discriminative features from raw processed signals (8, 8). Convolutional neural networks (CNNs), in particular, have demonstrated significant promise in extracting spatial and spectral patterns from EEG data, outperforming traditional methods in applications such as emotion recognition and seizure detection. However, existing deep learning models for EEG-based depression screening often fail to explicitly prioritize the subtle gradient and edge-like information in EEG signals—features that are increasingly recognized as critical to identifying depression-related biomarkers, such as localized aberrations in neural synchrony. While some studies have employed Sobel operators (9) as a preprocessing step to enhance edge features, these methods typically treat gradient extraction as a fixed, offline process (10). This disconnects edge detection from the subsequent learning stages and limits the model’s ability to adapt to depression-specific patterns.

To address this gap, we propose the Sobel Network, a novel neural architecture that intrinsically integrates gradient-based feature learning within its convolutional layers rather than treating edge detection as a separate preprocessing step. By embedding Sobel-inspired gradient operations directly into the trainable convolutional structure, the model is uniquely positioned to emphasize edge-like transitions and spatial gradients—features that align with known neurophysiological biomarkers of depression in EEG signals.

This end-to-end integration enables the network to dynamically adapt and prioritize depression-relevant patterns during training, thereby overcoming the static limitations of traditional preprocessing-based approaches.

The main contributions of this work are as follows:

1. A novel neural architecture, the Sobel Network, is proposed, which intrinsically integrates Sobel-inspired gradient operations into convolutional layers, enabling end-to-end learning. It can emphasize gradient patterns and edge-like information that are highly relevant to depression biomarkers in EEG, breaking through the limitation of previous approaches that solely used Sobel operators for preprocessing.
2. Evaluations on relevant datasets show that this architecture significantly outperforms other deep learning models in key metrics such as accuracy (reaching 98.67%), sensitivity, and specificity. The Sobel Network provides a promising avenue to improve the accuracy and robustness of automated EEG-based depression screening tools and has practical impact on clinical decision support.

## 2 Methods

### 2.1 Datasets and experiment setup

This study utilized an EEG dataset acquired at the Hospital of Universiti Sains Malaysia, comprising 34 patients diagnosed with major depressive disorder (MDD; 17 men; mean age, 40.3 ± 12.9 years) and 30 healthy controls (HC; 21 men; mean age, 38.2 ± 15.6 years) (11). Each participant completed 5 min of resting-state EEG under both eyes-closed and eyes-open conditions, recorded from 20 scalp electrodes (Fp1, Fp2, F3, F4, F7, T3, T5, C3, C4, Fz, Cz, Pz, F8, T4, T6, P3, P4, O1, O2, ECG) arranged according to the international 10–20 system at a sampling rate of 256 Hz, where the EEG data were recorded with Linked Ear (LE) reference and were re-referenced to the Infinity Reference (IR) (11). The exclusion criteria included psychosis, pregnancy, substance abuse, smoking, and epilepsy. The control subjects were free from any neurological or psychiatric disorders. The EEG signals were preprocessed using BESA software (11, 12) to remove artifacts, which included the application of a 50-Hz notch filter to eliminate power line interference. From the cleaned data, 2-min artifact-free segments were extracted per condition using a 4-s sliding window, resulting in a total of 18,442 epochs (9,789 from MDD subjects and 8,653 from controls).

Experiments were conducted using a standardized computing environment: a desktop equipped with an Intel i7 processor (3.33 GHz), 64 GB of RAM, an Nvidia RTX4090 graphics card with 24 GB memory, and operating on a 64-bit Windows 10 system with Keras of 2.10.0.

### 2.2 Definition of EEG edge features

In the context of this study, edge features in EEG signals refer to spatially localized abrupt transitions or gradients observed in multi-channel topographic maps. The Sobel Network processes these EEG edge features by adaptively responding to data variations across different spatial locations, enabling it to capture depression-relevant neural patterns with enhanced precision. These include the following:

- Phase-based edges: Sudden discontinuities in phase synchrony between adjacent brain regions, potentially indicating disruptions in functional connectivity networks
- Energy/amplitude gradients: Sharp spatial variations in signal power or amplitude across electrode arrays, possibly reflecting localized abnormalities in neural activity
- Spectral boundary features: Rapid transitions in frequency-specific patterns across cortical regions, which may correspond to pathological changes in oscillatory dynamics

These edge characteristics are particularly relevant for depression identification as they may capture the breakdown in normal large-scale neural coordination observed in MDD patients. The Sobel operator’s sensitivity to spatial gradients makes it particularly suitable to amplify these clinically relevant features while suppressing diffuse background activity.

### 2.3 Sobel edge detection layer

The Sobel edge detection layer is the core innovation of our model. It computes horizontal and vertical gradients of the input using fixed kernels. The horizontal gradient kernel is defined as Equation 1:

(1) $$G_{x}=\left[\begin{array}{ccc} -0.1 & 0 & 0.1\\ -0.2 & 0 & 0.2\\ -0.1 & 0 & 0.1 \end{array}\right],$$

and the vertical gradient kernel define in Equation 2

(2) $$G_{y}=\left[\begin{array}{ccc} -0.1 & -0.2 & -0.1\\ 0 & 0 & 0\\ 0.1 & 0.2 & 0.1 \end{array}\right].$$

Given an input tensor $X\in {\mathbb{R}}^{B\times H\times W\times C}$, where B is the batch size, H×W is the spatial dimension, and C is the number of channels, we replicate and concatenate the Sobel kernels $N_{f}=10$ times to construct the filter bank Equation 3:

(3) $$F=\overset{Nf}{\underset{i=1}{⊕}}[G_{x}⊕G_{y}],$$

where ⊕ denotes channel-wise tensor concatenation. The convolution operation is defined as Equation 4:

(4) $$\nabla X=\text{conv}2\text{d}\left(X,F,\;\text{stride}=1,\;\text{padding}{=}^{\prime }SAME^{\prime }\right),$$

resulting in an output $\nabla X\in {\mathbb{R}}^{B\times H\times W\times N_{f}}$, which captures directional edge features at each location.

The convolutional block consists of two layers. The first convolutional layer applies 20 filters of size 2 × 9, using a ReLU activation function Equation 5:

(5) $$C_{1}=\text{ReLU}\left(\text{conv}2\text{d}\left(\nabla X,W_{1}\right)\right),$$

which outputs a feature map of size (H−1)×(W−8)×20. The second convolutional layer applies 18 filters of size 8×7, with no activation (Equation 6):

(6) $$C_{2}=\text{ conv}2\text{d}\left(C_{1},W_{2}\right),$$

yielding an output of size (H−8)×(W−14)×18.

The fully connected classification head begins with flattening the output Equation 7:

(7) $$F=\text{flatten}\left(C_{2}\right),\;F\in {\mathbb{R}}^{D_{f}},\;D_{f}=\left(H-8\right)\left(W-14\right)\cdot 18.$$

This is followed by two dense (Equations 8, 9) layers and a final sigmoid output (Equation 10):

(8) $$H_{1}=\sigma \left(W_{3}\cdot F+b_{3}\right),\;\;H_{1}\in {\mathbb{R}}^{350},$$

(9) $$H_{2}=W_{4}\cdot H_{1}+b_{4},\;H_{2}\in {\mathbb{R}}^{60},$$

(10) $$\hat{y}=\sigma \left(W_{5}\cdot H_{2}+b_{5}\right),\;\;\hat{y}\in {\mathbb{R}}^{1},$$

where $\sigma \left(x\right)=\frac{1}{1+e^{-x}}$ is the sigmoid activation function.

The model is trained using a mean squared error (MSE) loss function Equation 11:

(11) $$ℒ=\frac{1}{B}\sum_{i=1}^{B}{\left(y_{i}-{\hat{y}}_{i}\right)}^{2},$$

where B is the batch size, and $y_{i}$ and ${\hat{y}}_{i}$ denote the true and predicted labels, respectively. Optimization is performed using stochastic gradient descent (SGD) with momentum Equations 12 and 13:

(12) $$v_{t}=\gamma v_{t-1}+\eta \nabla_{\theta }ℒ\left(\theta_{t}\right),$$

(13) $$\theta_{t+1}=\theta_{t}-v_{t},$$

with learning rate η=0.01, momentum γ=0.9, and Nesterov acceleration enabled.

To mitigate overfitting, a dropout layer is applied after the input, with a drop probability of 0.03 Equation 14:

(14) $$X_{\text{drop}}=\text{Dropout}\left(X,\;p=0.03\right).$$

This architecture effectively combines edge-preserving gradient filtering with convolutional representation learning, enabling robust classification under noisy conditions.

## 3 Results

### 3.1 Feature enhancement and robustness validation experiment based on Sobel layer

This experiment was designed to assess the noise robustness of our convolutional neural network in an EEG classification task and to quantify the signal enhancement effect introduced by the Sobel layer. EEG recordings are typically contaminated by electromyographic and electrooculographic artifacts, which degrade both classification accuracy and signal quality; thus, methods that effectively increase the signal-to-noise ratio (SNR) are essential. We propose inserting a Sobel edge-detection filter layer (Sobel layer) at the network’s input to extract salient spatial features and suppress noise. The network outputs both the classification prediction and the Sobel-processed signal, enabling computation of signal-quality metrics (e.g., improvements in SNR, PSNR, and SSIM). By systematically varying the input noise level (testing across multiple SNR conditions), we evaluated the model’s classification performance and signal enhancement capability, thereby demonstrating enhanced noise robustness for EEG classification.

**Figure 1:**
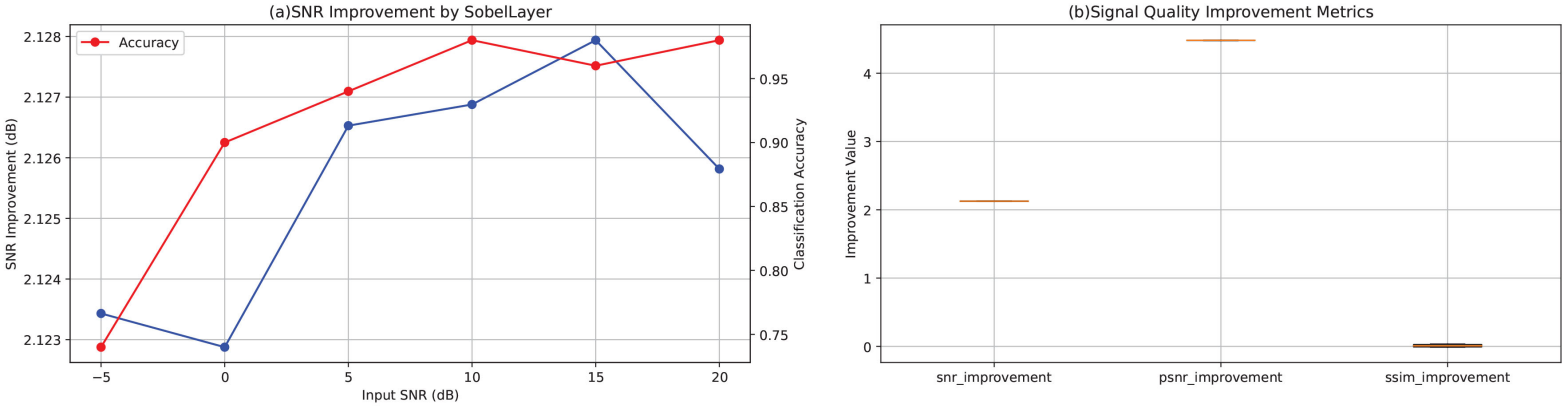
**(a)** SNR improvement (blue curve) introduced by the Sobel layer under different input SNR conditions and the corresponding classification accuracy (red curve, secondary axis) as a function of input SNR, averaged across all test samples and channels. **(b)** Boxplots of signal quality enhancement metrics across all test samples, including improvements in SNR, PSNR, and SSIM.

**Figure 1a** plots the SNR improvement conferred by the Sobel layer (blue curve) and the corresponding classification accuracy (red curve, secondary axis) as functions of input SNR, based on aggregate results from all test samples and channels. Despite a reduction in input SNR from 20 dB to −5 dB, the Sobel layer consistently yields an output SNR gain of approximately 2.12–2.13 dB—for example, an input SNR of 17.98 dB (nominally 20 dB) is raised to 20.11 dB (*a* = 2.13 dB), and an input SNR of −7.03 dB (nominally − dB) is elevated to − 4.91 dB (Δ ≈ 2.12 dB), demonstrating strong resilience under high-noise conditions. Classification accuracy remains relatively stable but shows a declining trend as noise increases: from 0.98 at 20 dB to 0.96 at 15 dB and 0.98 at 10 dB, then decreasing to 0.94, 0.90, and 0.74 at 5, 0, and −5 dB respectively, indicating that extreme noise still impacts classification performance.

**Figure 1b** presents boxplots of the three enhancement metrics (SNR, PSNR, and SSIM) computed across all test samples. SNR improvements are tightly clustered approximately 2.12–2.13 dB, and PSNR gains approximately 4.48 dB, indicating stable enhancement with minimal variability. SSIM changes average near zero: slightly negative at high SNR conditions (e.g., −0.0095 at 20 dB and −0.0117 at 15 dB) and slightly positive under low SNR conditions (e.g., +0.0335 at 0 dB and +0.0291 at −5 dB), suggesting that under heavy noise, the Sobel layer aids in recovering structural information.

The consistent performance across all SNR conditions and the minimal variability in enhancement metrics demonstrate that the Sobel layer provides robust feature enhancement throughout the entire recording duration, validating its effectiveness for EEG-based depression screening under realistic noisy conditions.

### 3.2 Performance on identifying MDD

We first conducted a parameter sensitivity analysis on the number of Sobel filters. Without loss of generality, we monitored the classification performance when the number of filters was set to 10, 20, and 30, which yielded results of 98.72%, 98.67%, and 98.67%, respectively. The classification results indicate that the number of filters has no direct correlation with the classification performance, and the optimal setting is 10.

**Figure 2:**
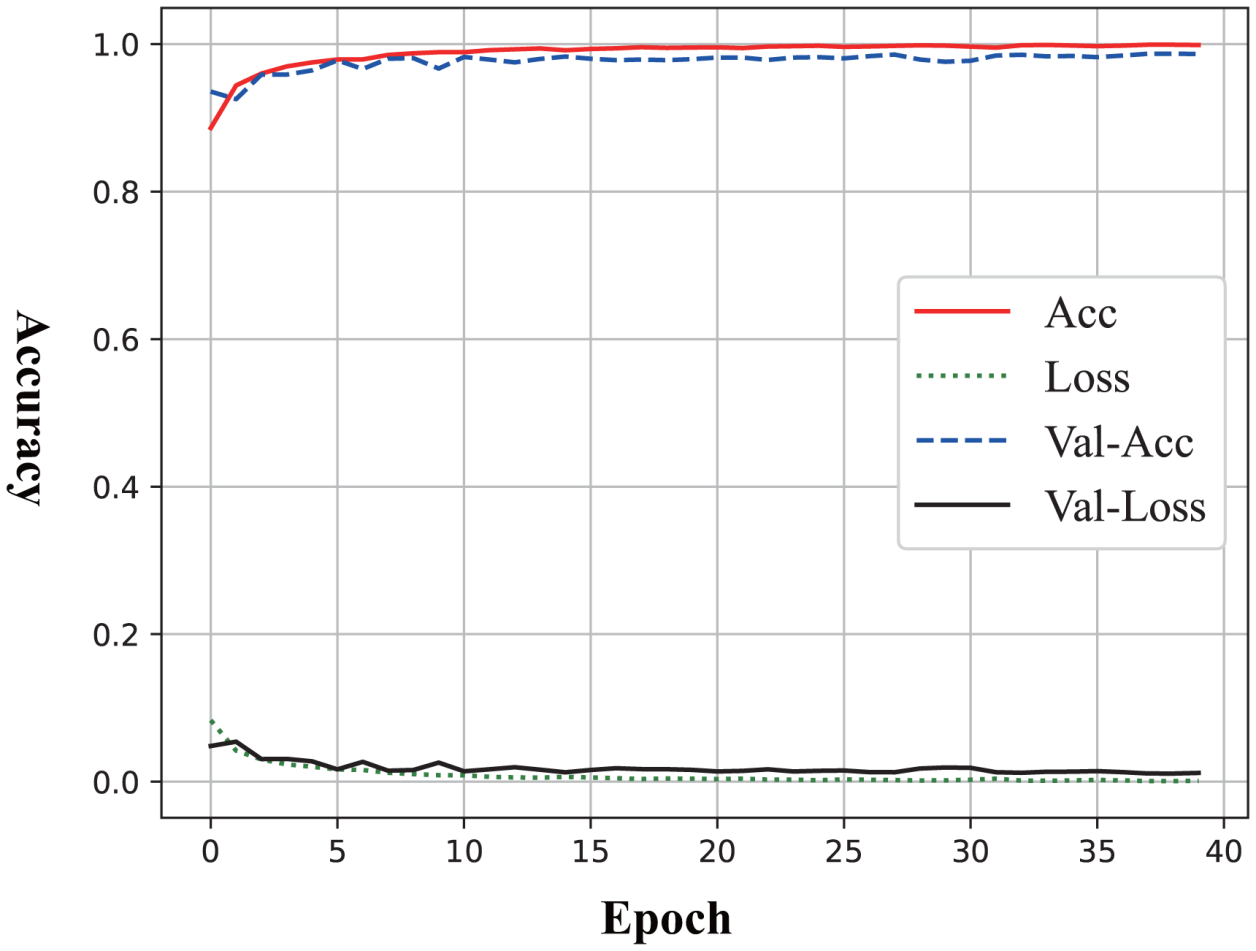
Learning curve of Sobel Network on MDD identification.

The learning curve presented in **Figure 2** illustrated the performance trends of the model during the training process to identify MDD, with the x-axis representing the number of training epochs (ranging from 0 to 40) and the y-axis indicating the metric values (ranging from 0.0 to 1.0). From the curve, the overfitting and underfitting did not occur because the training accuracy and validation accuracy exhibit a “U-shaped” pattern while improving synchronously.

The performance is evaluated based on a series of quantitative metrics such as accuracy, sensitivity, and specificity, with comparisons between different models and existing state-of-the-art methods. The results aim to validate the effectiveness of the proposed framework in improving MDD identification accuracy, especially in capturing the subtle neurophysiological patterns associated with MDD. **Table 1** compares the performance of five approaches in terms of sensitivity, specificity, and accuracy. Among them, the Sobel Network proposed in this paper ranks first with 98.56% specificity and 98.72% accuracy, while the TahnReLU-based CNN exhibits slightly superior sensitivity (99.03%). Both approaches significantly surpass other methods (e.g., GoogLeNet: 93.74% accuracy; Resnet-16: only 82.26%), indicating that the newly proposed Sobel Network demonstrates optimal comprehensive recognition capabilities—particularly excelling in positive sample detection—whereas the TahnReLU-based CNN shows marginally better robustness in discriminating negative samples. Both are suitable for high-precision scenarios. Possible reasons include the following: (1) The Sobel Network integrates traditional Sobel edge detection operators to enhance critical feature extraction, significantly improving sensitivity in identifying subtle structures (e.g., lesion boundaries in medical images), thereby optimizing positive sample detection while maintaining high specificity through end-to-end deep learning training and (2) The TahnReLU-based CNN leverages an improved activation function design (combining Tanh and ReLU), which mitigates ReLU’s neuron death issue while enhancing robustness in negative sample discrimination via Tanh’s saturation characteristics, achieving exceptional performance in balanced inter-class recognition.

**Table 1:** Detailed description.

Approach	Sensitivity	Specificity	Accuracy
GoogLeNet (13)	96.48	90.62	93.74
Resnet-16 (13)	88.9	74.79	82.26
MLRW (11)	95	80	87.5
TahnReLU-based CNN (14)	99.03	98.21	98.64
Sobel Network	98.87	98.56	98.72

## 4 Discussions and conclusions

Sobel Network to capture electrophysiological signatures of depression: The success of the Sobel Network in capturing electrophysiological signatures of brain dysfunction in depression can be attributed to its unique ability to emphasize spatial gradient and edge-like features in EEG signals, which are known to reflect key neuropathological mechanisms of MDD. Depression-related abnormalities—including altered frontal alpha asymmetry, disrupted functional connectivity, and aberrant neural synchronization—often manifest as subtle spatial gradients and edge-like transition patterns in multi-channel EEG topography. The Sobel Network, through its embedded gradient operations, dynamically enhances these fine-grained spatial variations, thereby effectively capturing neural circuit abnormalities associated with depression.

Sobel-based EEG enhancement for noise-robust classification: The results show that adding a Sobel layer consistently improves SNR and PSNR across noise conditions, with minimal impact on SSIM. Classification accuracy declines as noise rises, as expected. Quantitatively, the Sobel layer provides average gains of 2.125 dB in SNR and 4.48 dB in PSNR, highlighting its ability to enhance signal features while suppressing noise. The near-zero SSIM changes (slight decrease at high SNR) may stem from Sobel’s edge emphasis, introducing structural variations irrelevant to classification. Performance stays high at 20 dB (98%) but drops under severe noise, emphasizing remaining challenges in extreme cases. Overall, the Sobel layer is an effective feature enhancement module for EEG networks, improving signal quality and noise robustness. Future work may integrate advanced denoising or multi-channel fusion to boost performance in adverse conditions.

Fusion of signal processing and deep learning: The deep integration of traditional signal processing methods with neural networks (15) has shown significant benefits in EEG-based depression screening, with broader relevance to biomedical signal analysis and cross-domain data processing. This approach bridges prior knowledge from traditional techniques with the end-to-end learning capability of neural networks, mitigating deep learning’s heavy data dependence and “black box” limitations while overcoming traditional methods’ constraints in modeling complex patterns (16). This integrative strategy also applies effectively to wavelet transform, a classical time–frequency analysis tool adept at decomposing non-stationary signals (e.g., EEG, EMG, ECG) and capturing transient changes and local energy fluctuations—features often critical to identifying abnormal physiological states. However, traditional wavelet-based feature extraction relies on manual design and lacks end-to-end optimizability with classifiers. By embedding wavelet multi-scale decomposition into neural networks [e.g., via Fourier (17) or wavelet attention mechanisms (18)], models can autonomously learn discriminative time–frequency features—such as abnormal EEG delta waves or fMRI BOLD fluctuations—and achieve joint optimization of feature extraction and classification through end-to-end training.

Extend applications: The proposed hybrid framework, which leverages the strengths of both traditional signal processing and deep learning, is not limited to EEG signal enhancement. Its philosophy of combining domain-knowledge-driven preprocessing with data-driven modeling holds significant potential to inspire solutions in a wider range of cross-domain applications—for instance, similar frameworks could be explored for deep-learning-based intelligent vehicle control (19), where traditional control theory meets neural networks, or for complex multimodal fusion tasks (20) that require robust feature extraction from heterogeneous data sources. Furthermore, the principles could be adapted to advance unsupervised learning paradigms (21) by integrating structured prior knowledge to guide the learning process.

Future works: To tackle the issue of generalizability, we will conduct extensive cross-dataset validation utilizing several publicly available EEG datasets to thoroughly assess the transferability of our model. Concurrently, to bridge the gap between artificial and real noise, we are designing experiments to incorporate EEG data with naturally occurring artifacts. This will involve a systematic analysis of the model’s performance on real-world noisy data and a discussion on its practical robustness for clinical application.

Conclusions: This study proposes and validates the Sobel Network—an innovative neural architecture that embeds Sobel gradient operators for end-to-end learning—designed for EEG-based depression screening. Experiments demonstrate that the model significantly outperforms existing methods in key metrics (accuracy: 98.67%, sensitivity: 99.18%). Its core advantages stem from (1) a trainable Sobel layer that dynamically enhances edge-gradient features in EEG signals (highly correlated with depression biomarkers), maintaining a stable SNR gain of 2.125 dB under noisy conditions and (2) a groundbreaking fusion of traditional signal-processing priors with deep learning adaptability, overcoming limitations of decoupled preprocessing approaches.

This “domain-knowledge-embedded” paradigm not only provides a high-precision tool for objective depression screening but also pioneers new pathways for cross-modal biomedical signal analysis (e.g., wavelet neural network fusion), advancing the development of interpretable, low-data-dependent intelligent diagnostic systems.

## Data Availability

The original contributions presented in the study are included in the article/supplementary material. Further inquiries can be directed to the corresponding authors.
